# Microarray expression profiling in the denervated hippocampus identifies long noncoding RNAs functionally involved in neurogenesis

**DOI:** 10.1186/s12867-017-0091-2

**Published:** 2017-06-06

**Authors:** Bingying Deng, Xiang Cheng, Haoming Li, Jianbing Qin, Meiling Tian, Guohua Jin

**Affiliations:** 1 Department of Anatomy and Neurobiology, The Jiangsu Key Laboratory of Neuroregeneration, Co-innovation Center of Neuroregeneration, Nantong University, Nantong, 226001 Jiangsu People’s Republic of China; 2 Medical School of Nantong University, Building 3, No. 19 Qixiu Road, Congchuan District, Room 325, Nantong, 226001 China

**Keywords:** Hippocampus, Long noncoding RNA, Microarray, Neural stem cells, Neurogenesis

## Abstract

**Background:**

The denervated hippocampus provides a proper microenvironment for the survival and neuronal differentiation of neural progenitors. While thousands of lncRNAs were identified, only a few lncRNAs that regulate neurogenesis in the hippocampus are reported. The present study aimed to perform microarray expression profiling to identify long noncoding RNAs (lncRNAs) that might participate in the hippocampal neurogenesis, and investigate the potential roles of identified lncRNAs in the hippocampal neurogenesis.

**Results:**

In this study, the profiling suggested that 74 activated and 29 repressed (|log fold-change|>1.5) lncRNAs were differentially expressed between the denervated and the normal hippocampi. Furthermore, differentially expressed lncRNAs associated with neurogenesis were found. According to the tissue-specific expression profiles, and a novel lncRNA (lncRNA2393) was identified as a neural regulator in the hippocampus in this study. The expression of lncRNA2393 was activated in the denervated hippocampus. FISH showed lncRNA2393 specially existed in the subgranular zone of the dentate gyrus in the hippocampus and in the cytoplasm of neural stem cells (NSCs). The knockdown of lncRNA2393 depletes the EdU-positive NSCs. Besides, the increased expression of lncRNA2393 was found to be triggered by the change in the microenvironment.

**Conclusion:**

We concluded that expression changes of lncRNAs exists in the microenvironment of denervated hippocampus, of which promotes hippocampal neurogenesis. The identified lncRNA lncRNA2393 expressed in neural stem cells, located in the subgranular zone of the dentate gyrus, which can promote NSCs proliferation in vitro. Therefore, the question is exactly which part of the denervated hippocampus induced the expression of lncRNA2393. Further studies should aim to explore the exact molecular mechanism behind the expression of lncRNA2393 in the hippocampus, to lay the foundation for the clinical application of NSCs in treating diseases of the central nervous system.

**Electronic supplementary material:**

The online version of this article (doi:10.1186/s12867-017-0091-2) contains supplementary material, which is available to authorized users.

## Background

The mammalian brain is the most complex organ among all living organisms. This enormous complexity is generated via proliferation and differentiation of multipotent neural stem cells (NSCs) into multiple cell types. Researchers have demonstrated life-long continuous neurogenesis in almost all the mammals, including humans [1]. The main neurogenic regions in the adult murine brain are the subgranular zone of dentate gyrus (DG) in the hippocampus and the subependymal zone of lateral ventricles, also called ventricular–subventricular zone (V-SVZ) [2–6]. In the adult brain, the hippocampus is a crucial structure for the formation of certain types of memory, such as episodic memory and spatial memory [7]. Meanwhile, emerging data have implied that hippocampal neurogenesis can lead to improvement in therapies for neurological disorders, including cerebral ischemia, depression, Alzheimer’s disease, epilepsy, and Parkinson’s disease, many of which are associated with cognitive decline [8]. Thus, researchers are highly interested in exploring how various developmental events associated with hippocampal neurogenesis are regulated.

So far, it was well accepted that the hippocampal neurogenesis is under the control of gene regulatory network, especially transcription factors, microRNAs (miRNAs), and signaling pathways [9, 10]. However, annotation and high-throughput deep sequencing of transcriptomes have revolutionized the view previously held for the mammalian genome. Surprisingly, a major part of the genome is transcribed into long noncoding RNAs (lncRNAs), which are longer than 200 nucleotides (nt) in length and lack an open reading frame in sequence [11, 12]. Increasing evidence indicated that lncRNAs participate in gene regulatory networks controlling the development and functioning of various tissues [12–15]. Moreover, transcript expression analyses within the nervous system have shown an abundance of lncRNAs that display spatially restricted and temporally dynamic expression [16–19].

Hence, the aim of the present study was to perform microarray expression profiling to identify lncRNAs that might participate in the hippocampal neurogenesis. A set of lncRNAs differentially expressed in the hippocampus after fimbria-fornix (FF) transection were identified. A previous study found that the internal microenvironment changed after FF transection and subsequently contributed to the migration and survival of transplanted and endogenous hippocampal NSCs [20]. Moreover, this study indicated a relationship between dysregulated lncRNAs and the changes in the hippocampal environment. These findings may be helpful in understanding the role of the novel lncRNAs in hippocampal neurogenesis. Overall, the present study demonstrated that an evolutionarily conserved lncRNA regulated neurogenesis from NSCs in the embryonic brain.

## Methods

### Animals

40 adult Sprague–Dawley rats (23 male and 17 female) and 4 E17 Sprague–Dawley rat were used in this study. All animals used in the present study were provided by the Experimental Animal Centre of Nantong University, China. The experimental procedures involving animals were approved by Jiangsu Institutes of Health Guide for the Care and Use of Laboratory Animals. All efforts were made to minimize the number and suffering of animals used in the study, and all the experiments were repeated several times to minimize the experimental error. The rats were housed in a temperature-controlled room at 23 ± 2 °C maintained on a 12-h light/12-h dark cycle, and caged in a facility where food and water were available ad libitum. The rats were anesthetized with chloral hydrate (2 mL/kg body weight) to change the microenvironment in the hippocampus, the transection of FF was performed with a wire knife at the CA1 layer of the dorsal hippocampus, at coordinates of bregma: anteroposterior 1.4 mm; lateral 1–4 mm; depth 5.6 mm. There is no restriction on the gender of the animals.

### Microarray

RNA samples were extracted from the paired rat untreated hippocampus 7 days after the FF transection. The hippocampal tissue (denervated and untreated) from three paired Sprague–Dawley rats (2 male and 1 female) was quickly harvested on the ice. For distant shipping, all the tissues were immediately frozen in liquid nitrogen. Total RNA was then extracted with TRIzol reagent following the manufacturer’s instruction. The RNA integrity number (RIN) was evaluated to judge the integrity of RNA samples using Agilent 2200 Bioanalyzer (Agilent Technologies, USA) following the manufacturer’s protocol [23]. The purity of RNA samples was evaluated by agarose gel electrophoresis and ultraviolet spectrophotometer K5500 (Beijing Kaiao Technology Development Co., Ltd, China). A260/A280 ≥ 1.5 and A260/A230 ≥ 1 indicated acceptable RNA purity, and RIN value ≥7 using the Agilent 2200 RNA assay indicated acceptable RNA integrity (Additional file 1: Table S1). Genomic DNA contamination was evaluated by gel electrophoresis (Additional file 1: Figure S1). Three independent samples were assayed to evaluate the reproducibility of the experimental procedure. Fluorescent complementary DNA (cDNA) was synthesized from about 2 µg of total RNA using an Amino Allyl MessageAmp II Kit (Life Technologies, USA). Hybridization with RiboArray™ lncDETECT™ RAT Array 1*12K was performed following the manufacturer’s instruction (Riobio, China). The slides were washed and then scanned and analyzed using a GenePix 4000B Microarray Scanner (Molecular Devices, USA). The microarray data was corrected by 50% scaling method to eliminate the system error of the experiment [21]. Fold change was calculated by 2-Fold change = log_2_ (normalized intensity of treat/normalized intensity of control). As to the same for the repeated probes in the same microarray, the median value is taken as the signal value of the probe. For the repeated probes in the different microarray from the same samples, the mean value is taken as the signal value of the probe. The *p* value was calculated by Product Rank statistical test method [22]. The differential gene expression in samples were calculated using limma package in Bioconductor. The genes which meet the condition that |2-Fold change|>1 and *p* value <0.05 was considered the differentially expressed genes. Pathway significant enrichment analysis was based on the KEGG Pathway Public database and found the significant enrichment pathway among the differential expressed genes applied the Hypergeometric test. With the Pathway analysis results, we can identify the main biological process and signal transduction pathways which the differential expressed genes involved.

### RNA extraction and quality control

Total RNA was extracted from NSCs and the hippocampus on different days after FF transection (1, 3 and 7 days), using TRIzol reagent (Invitrogen, USA) following the standard protocol. Individual tissues, namely striatum, hippocampus, brainstem, cerebellum, cerebrum, heart, pancreas, muscle, and liver, were dissected; the utmost care was taken to ward off contamination. The tissues were washed in phosphate-buffered saline (PBS) several times to clean up the debris. All the RNA samples were under the strict quality control. Quantification and quality check were performed using Nanodrop 2000 (Thermo Scientific, USA). LncRNAs were reverse transcribed using a RevertAid First Strand cDNA Synthesis Kit (Thermo Scientific) at 65 °C for 5 min, 42 °C for 60 min, and 72 °C for 5 min.

### Quantitative real-time polymerase chain reaction and semi-quantitative PCR

The primers used for polymerase chain reaction (PCR) were designed and synthesized by RiboBio (Guangdong, China). The intellectual property rights of the primer sequence belonged to Ribo biology, which were asked to be classified. Quantitative real-time PCR and semi-quantitative PCR were conducted using SYBR Green Master Mix (Roche, Germany) and Dream Taq Green PCR Master Mix (Thermo Scientific), respectively. The reactions were carried out using the Rotor Gene 6000 (Corbett Life Science, Australia) and Gene Amp PCR System 9700 (Corbett Life Science) with a 15-s initial denaturation step at 95 °C and 40 cycles of a 40-s denaturation step at 95 °C followed by a 40-s hybridization at 59 °C, ending with a melting curve analysis. Glyceraldehyde-3-phosphate dehydrogenase (GAPDH) and U6 were used as endogenous controls. Fold changes were calculated using the relative quantification 2^−ΔΔCt^ method.

### Hippocampal NSCs culture

4 Sprague–Dawley rat hippocampi (embryonic days 16–17) were used to derive NSC cultures. The cells were filtered through a 40-μm cell strainer (Biologix Research Company, USA). They were cultured at a density of 1 × 10^5^ cells/mL in an NSC self-renewal medium (Dulbecco’s modified Eagle’s medium, DMEM) with 2% B27 (Gibco Life Technologies, USA), 20 ng/mL Epidermal Growth Factor (EGF) (Sigma, USA), and 20 ng/mL basic Fibroblast Growth Factor (bFGF) (Sigma) at 37 °C and 5% CO_2_. The cells were passaged one or two generations to generated stable NSCs lines. The protocol of establishment neural stem cell cultures was successful and mature. For PCR, transfection, 5-ethynyl-2′-deoxyuridine (EdU) assay, and flow cytometry, 1 × 10^6^ cells/mL were cultivated on 6-well plates. For fluorescence in situ hybridization (FISH), 1 × 10^5^ cells/mL were cultivated on 24-well plates.

### Fluorescence in situ hybridization

Branched DNA FISH was performed in vivo in an adult rat coronal brain section and in vitro in rat NSCs, using a FISH kit purchased from RiboBio (Guangdong, China) following the manufacturer’s protocol. After washing twice with PBS, the section and cells were fixed in 4% paraformaldehyde for 30 min and then incubated in PBS with 0.1% Triton X-100 for 15 min. The hybridization mixed probe should be pre-heat first. The intellectual property rights of the primer sequence belonged to Ribo biology, which were asked to be classified. Before hybridization overnight at 4 °C, the samples were incubated in the prehybridization solution for 2 h at room temperature. On the second day, the cells were stained with Hoechst (Sigma, China) after washing at 42 °C in 4× saline sodium citrate (SSC) twice, 2× SSC once, 1 × SSC once, and 1 × PBS once. Then, samples were visualized using a confocal fluorescent microscope (Olympus, Japan). RNA isolated from nuclear and cytoplasmic fractions of neural stem cells. RNA isolation was carried out using a PARIS kit (Ambion, America). Neural stem cells were calculated and collected at most of 1 × 10^7^ cells after digested by trypsin. After washing cells with cold PBS, cells were resuspended in 500 μL ice-cold cell fractionation buffer for 10 min. After centrifuged samples 5 min at 4 ℃ and 500*g*, the supernatant cytoplasmic fraction were carefully collected with a micropipettor and nuclear fraction were the pellet at the bottom of the tube. Add 500 μL of ice-cold cell disruption buffer to the nuclear pellet, and make sure use a volume of cell disruption buffer equal to the volume of the cytoplasmic fraction to keep cytoplasmic and nuclear samples parallel. Mix the cytoplasmic and nuclear lysis/binding solution and pipet 3–4 times. Add 500 μL ACS grade 100% ethanol and mix gently. Draw the sample mixture through a filter cartridge, respectively. Wash once with 700 μL wash solution 1 and wash with 500 μL wash solution twice. At last, elute RNA with 40–60 μL of about 95 °C elution solution for the real-time PCR assay.

### Cell transfection

The transfection of NSCs was carried out using Lipofectamine 2000 (Invitrogen). Plasmid vectors and negative control for transfection were synthesized by RiboBio. The cells were transfected with a 100 nM plasmid vector using Lipofectamine 2000 following the manufacturer’s instructions. After transfection for 48 h, the expression levels of the selected lncRNAs were measured by qPCR. After 72 h, the cells were collected for cell proliferation assays.

### Cell proliferation assay

Neural stem cells proliferation was measured using the EdU (5-Ethynyl-2′-deoxyurdine) assay and flow cytometry. For the EdU assay, 1 × 10^5^ cells 72 h after transfection or nontransfected cells were suspended in serum-free DMEM containing 50 μL of EdU. After the cells were incubated in a 1.5-mL centrifuge tube for 2 h at 37 °C, they were mixed with 4% formaldehyde for 30 min at room temperature. After washing twice with 1 mL of PBS, EdU was detected with an Apollo 567 for 30 min at room temperature. Then, the cells were stained with Hoechst 33342 for 30 min and visualized using a fluorescent microscope (Olympus). The EdU incorporation rate was expressed as the ratio of EdU-positive cells (red cells) to total Hoechst 33342-positive cells (blue cells). For flow cytometry, the cells were fixed at 1 × 10^6^ cells/ml in the precooled 75% alcohol overnight after dispersion with trypsin (Sigma) and filtered through a 40-μm cell strainer. Then, the cells were harvested and stained with Annexin V-fluorescein isothiocyanate and propidium iodide (PI) (BD Biosciences, USA) for 30 min following the manufacturer’s instruction. The cells were analyzed using flow cytometry (BD Biosciences, USA). Data were analyzed using the CELL Quest 3.0 software. All experiments were performed in triplicate.

### Statistical analysis

Each finding was confirmed using three independent biological replicates, unless specified. All values were evaluated using the SPSS 18.0 statistical software (SPSS, IL, USA) and expressed as mean ± standard deviation. Statistical significance was calculated using the Student *t* test. A *p* value < 0.05 was considered statistically significant.

## Results

### Profile of microarray data

**Fig. 1:**
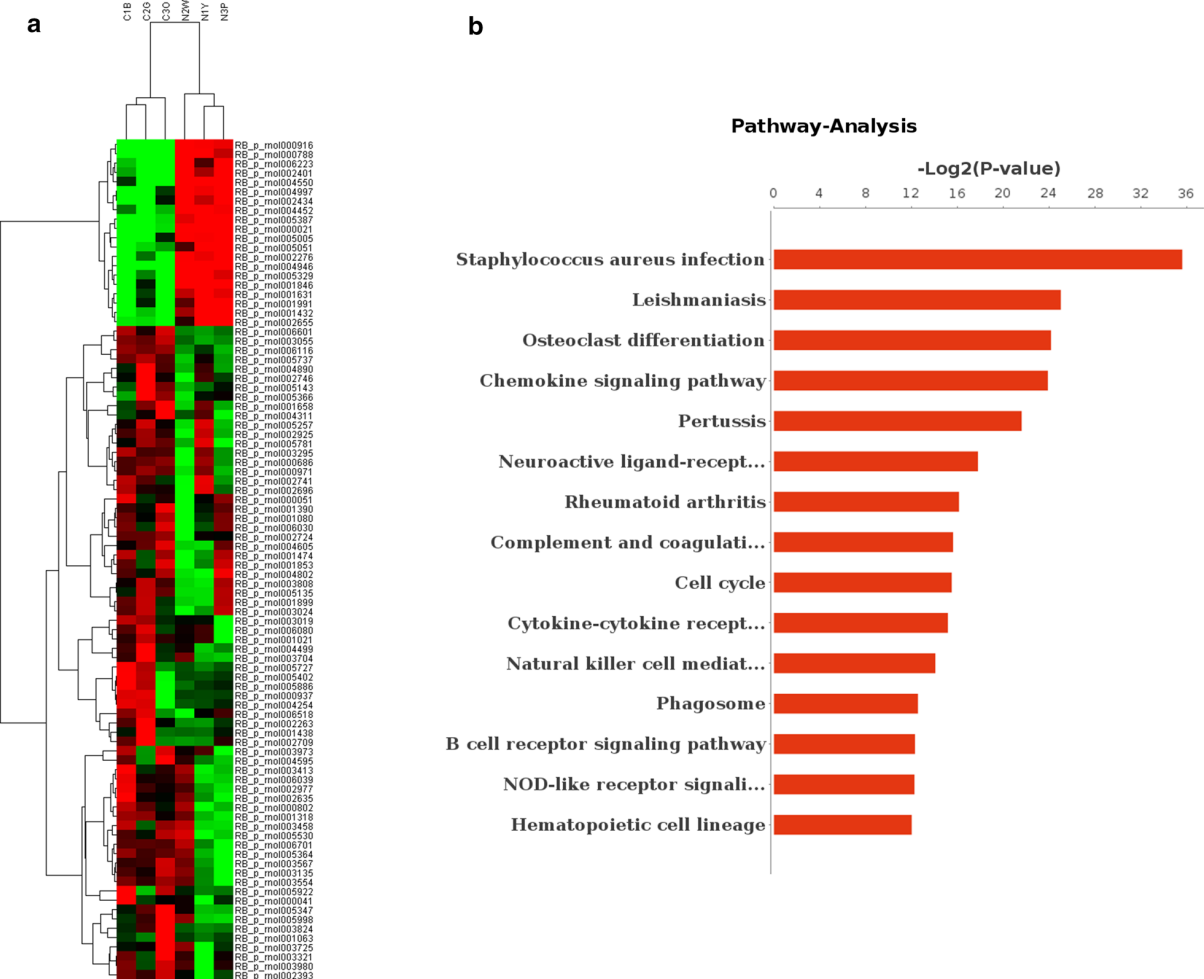
Clustering analysis and KEGG analysis of differential expressed lncRNAs and mRNAs, respectively. **a** Differentially expressed lncRNAs in the normal hippocampus and its paired denervated hippocampus analyzed using hierarchical clustering. Hierarchical clustering analysis arranges samples into groups by the expression level. *Red* means highly expressed, and *green* means lowly expressed. **b** KEGG analysis indicated that the differential expressed mRNAs after FF transaction were mainly involved the pathway about infection and cell cycle regulation

The microarray data are deposited in a public repository GEO, and the accession number is GSE96992, Using the microarray expression profiles, 103 (74 activated and 29 repressed) lncRNAs differentially expressed in the hippocampus after the FF transection were identified. The correlation of expression profiles between the biological replicates and the treatment conditions was demonstrated using unsupervised hierarchical clustering analysis (Fig. 1a). Pathway analysis was used to find out the significant pathway of the differential genes according to KEGG database. We turn to the Fisher’s exact test to select the significant pathway. Our results showed that * pathways were significantly enriched for the identified DEGs (p < 0.05). Moreover, pathway analysis showed that these genes were mainly involved in infection, cell cycle, and neurogenesis (Fig. 1b).

### Real-time quantitative PCR confirmation

A total of 20 lncRNAs with log fold-changes between 1 and 10 (17 upregulated and 3 downregulated) were randomly selected in different samples of the normal and paired denervated hippocampi to further validate the microarray results. The samples used for real-time PCR and microarray are the same ones. Each RNA samples of rat hippocampal tissue were divided into two parts, one for microarray and the other for real-time PCR.

**Fig. 2:**
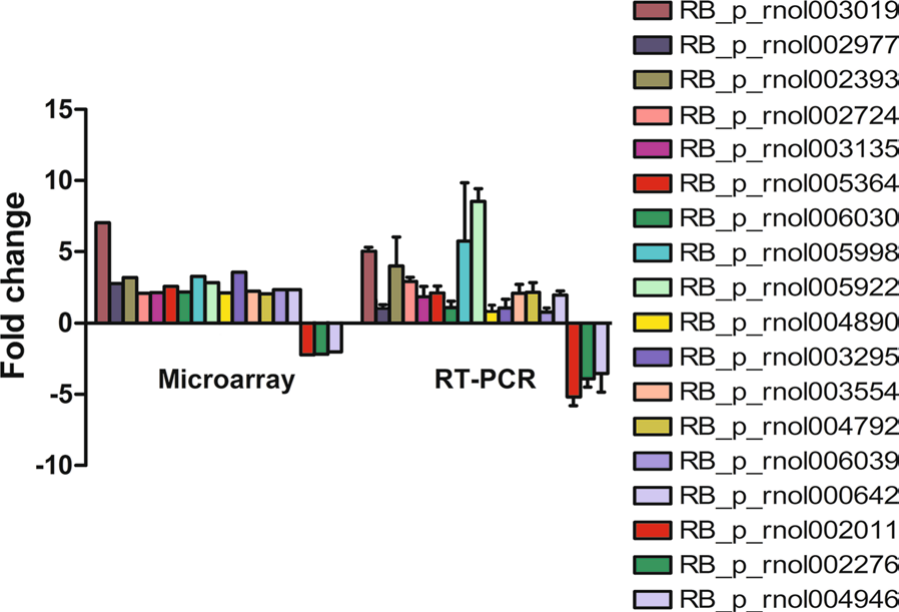
Validation of the expression of novel lncRNAs. The fold changes in the expression of lncRNAs on the seventh day after FF transection were normalized to the normal hippocampus. Results were based on average of three independent experiments (mean ± standard deviation). The fold changes in the expression of lncRNAs revealed by qPCR were consistent with the results from microarray expression profiling

All the selected lncRNAs are listed in Additional file 1: Table S1, including name and log fold-changes. Three pairs of primers were designed to certify the expression of lncRNAs. Only the primer whose PCR product was in accordance with the predicted nucleus base pair number was used for further studies. Using specific primers for each lncRNA, all candidate lncRNAs from hippocampal total RNA were PCR-amplified (Additional file 1: Figure S2). Among 20 candidates, two primers had no products. Using quantitative real-time reverse transcription-PCR (qPCR), the expression of these lncRNAs was found to be consistent with the microarray data (Fig. 2). Thus, these results further indicated the high accuracy of microarray expression profiles in detecting differential expression levels of most lncRNAs. Additionally, the microarray showed a series of lncRNAs constantly differentially expressed between the normal and paired denervated hippocampi.

### Expression signatures of differentially expressed lncRNAs

**Fig. 3:**
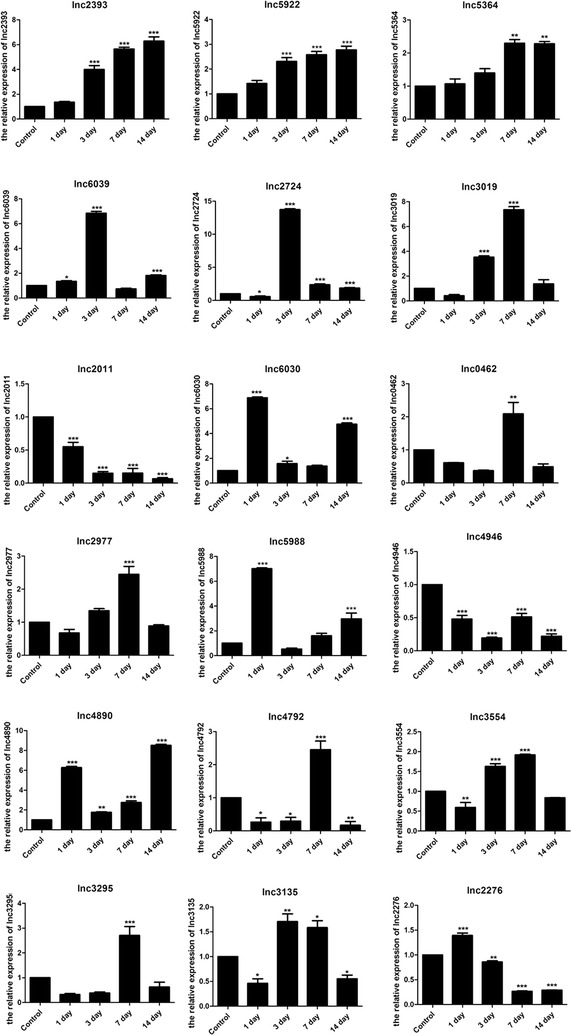
Expression signatures of differentially expressed lncRNAs in the hippocampus. The expression trend of 18 differentially expressed lncRNAs during 1, 3, 7 and 14 days after FF transfection. Results were based on average of three independent experiments (mean ± standard deviation). Statistical significance was calculated using Student *t* test (**p < 0.01, ***p < 0.001, compared to the control group)

Since transcript expression analyses have shown a number of lncRNAs that display restricted and temporally dynamic expression [12, 24, 25], the expression trend of 18 lncRNAs was first investigated during four different periods after FF transfection. The expression of lncRNAs extracted from the normal hippocampus and the hippocampi 1, 3, 7 and 14 days after FF transfection was testified. RT-qPCR demonstrated fluctuations in lncRNA expression after a change in the inner microenvironment. It was concluded that the expression levels of some lncRNAs (such as 5922, 5364, and 2393) continuously increased compared with the normal hippocampus, whereas the expression levels of some lncRNAs (such as 2011) always decreased. On the contrary, some lncRNAs (such as 6039, 2724, and 3019) showed no consistent changes instead of reaching a peak during one period (Fig. 3). The expression profiles of lncRNAs provided a hint of their potential functions during development.

**Fig. 4:**
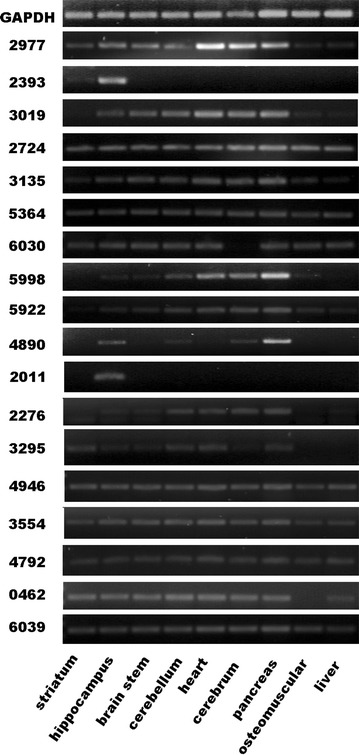
Tissue specialties of 18 candidate lncRNAs. RT-PCR revealed the 18 lncRNAs that exist in the following tissues: striatum, hippocampus, brain stem, cerebellum, cerebrum, heart, pancreas, muscle, and liver. Among 18 candidates, 2393 showed high specificity

lncRNAs generally show more tissue specificity compared with protein-coding genes [26]. The expression patterns of lncRNAs in different tissues (including striatum, hippocampus, brain stem, cerebellum, heart, pancreas, muscle, and liver) were identified, which were developed from three different germinal layers. RT-PCR was performed to further determine whether the candidate lncRNAs existed in some specific tissues or systems. It was found that some lncRNAs (2724, 5364, and 6039) were present in all aforementioned eight tissues, whereas some lncRNAs existed in the neural tissues and lineages (Fig. 4). Especially 2393 and 2011 lncRNAs showed high tissue specificity in the neural system, indicating their potential meaningful roles in neurogenesis.

A subsequent study to examine the differential expression levels of lncRNAs after FF transection and in various tissues using qPCR and RT-PCR led to the identification of lncRNA2393 that might participate in adult hippocampal neurogenesis.

**Fig. 5:**
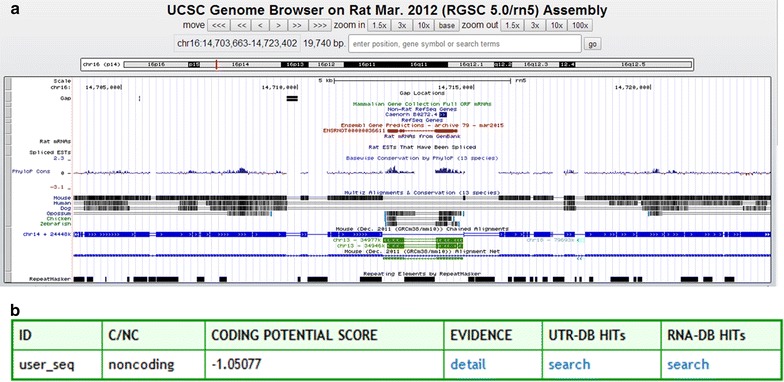
Information about the novel lncRNA2393. **a** UCSC showed lncRNA2393 location and homology with different species. **b** CPC demonstrated that lncRNA2393 had no potential of encoding protein

University of California Santa Cruz blast was used [27, 28]. To further identify the novel lncRNAs demonstrating lncRNA2393 to be a 961-nt polyadenylated RNA encoded by 8 exons (Fig. 5a). The analysis using the Coding Potential Calculator [29], PhyloCSF [30], and Coding-Potential Assessment Tool [31] indicated that the lncRNA2393 transcript had no protein-coding potential (Fig. 5b).

### Identification of the novel lncRNA in the hippocampus

**Fig. 6:**
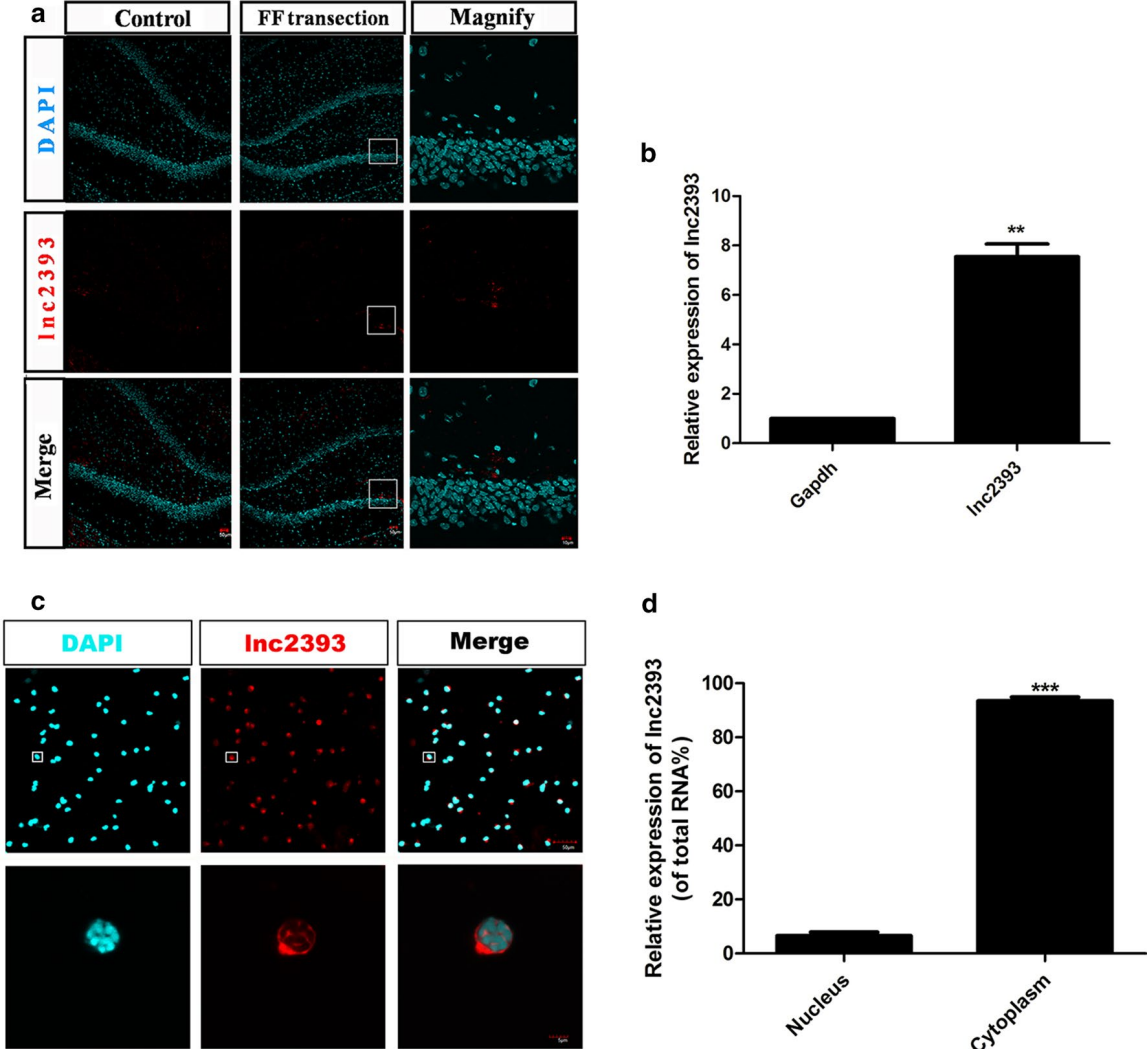
lncRNA2393 was expressed in hippocampus and V-SVZ NSCs. **a** FISH showed lncRNA2393 was expressed in the hippocampus and increased after FF transection. **b** RT-qPCR demonstrated the expression of lncRNA2393 in the NSCs (**p < 0.01). **c** FISH for lncRNA2393 in V-SVZ NSC cultures. Nuclei were counterstained with DAPI. **d** Subcellular fractionation followed by RT-qPCR for indicated lncRNAs. *Error bars* are propagated standard deviation from technical triplicate wells (***p < 0.001)

Throughout adult life, V-SVZ NSCs give rise to transit-amplifying cells, which generate neuroblasts that migrate to the olfactory bulb where they differentiate into interneurons [32–34]. It has been observed that FF transection would result in increasing neuronal production in the hippocampus. Hence, FISH was performed to explore the location of lncRNA2393 to more directly observe the expression trend and investigate the function of lncRNA2393, the DG or some other irrelevant location in the hippocampus. It was demonstrated that lncRNA2393 was specially expressed in the subgranular zone, which contained a population of adult NSCs (Fig. 6a). Compared with the normal hippocampus, the denervated hippocampus had more fluorescence-positive cells. Thus, it was hypothesized that the upregulated lncRNA2393 resulting from FF transection was correlated with the neurogenesis in the hippocampus. Total RNAs were prepared from NSCs that were extracted from the rat embryoid body To test and verify this assumption. As expected, the expression of lncRNA2393 was validated in the NSCs (Fig. 6b).

Considering the information on the subcellular localization of lncRNAs can provide an important hint about their possible function as a nuclear-restricted epigenetic regulator or cytoplasmic competing endogenous RNAs (ceRNAs) [35, 36], RT-qPCR of nuclear fractionation of hippocampal NSC cultures was performed. The analysis demonstrated lncRNA2393 to be enriched in the cytoplasm, compared with GAPDH, which is located in the cytoplasm (Fig. 6c). Consistent with the nuclear fractionation studies, FISH for lncRNA2393 demonstrated the predominantly cytoplasmic location of the transcript (Fig. 6d). Together, these data indicated that lncRNA2393 had high tissue specificity and tight transcriptional regulation, anticipating its potential role in the neurogenesis.

### LncRNA2393 silencing reduced the NSC proliferation ability

**Fig. 7:**
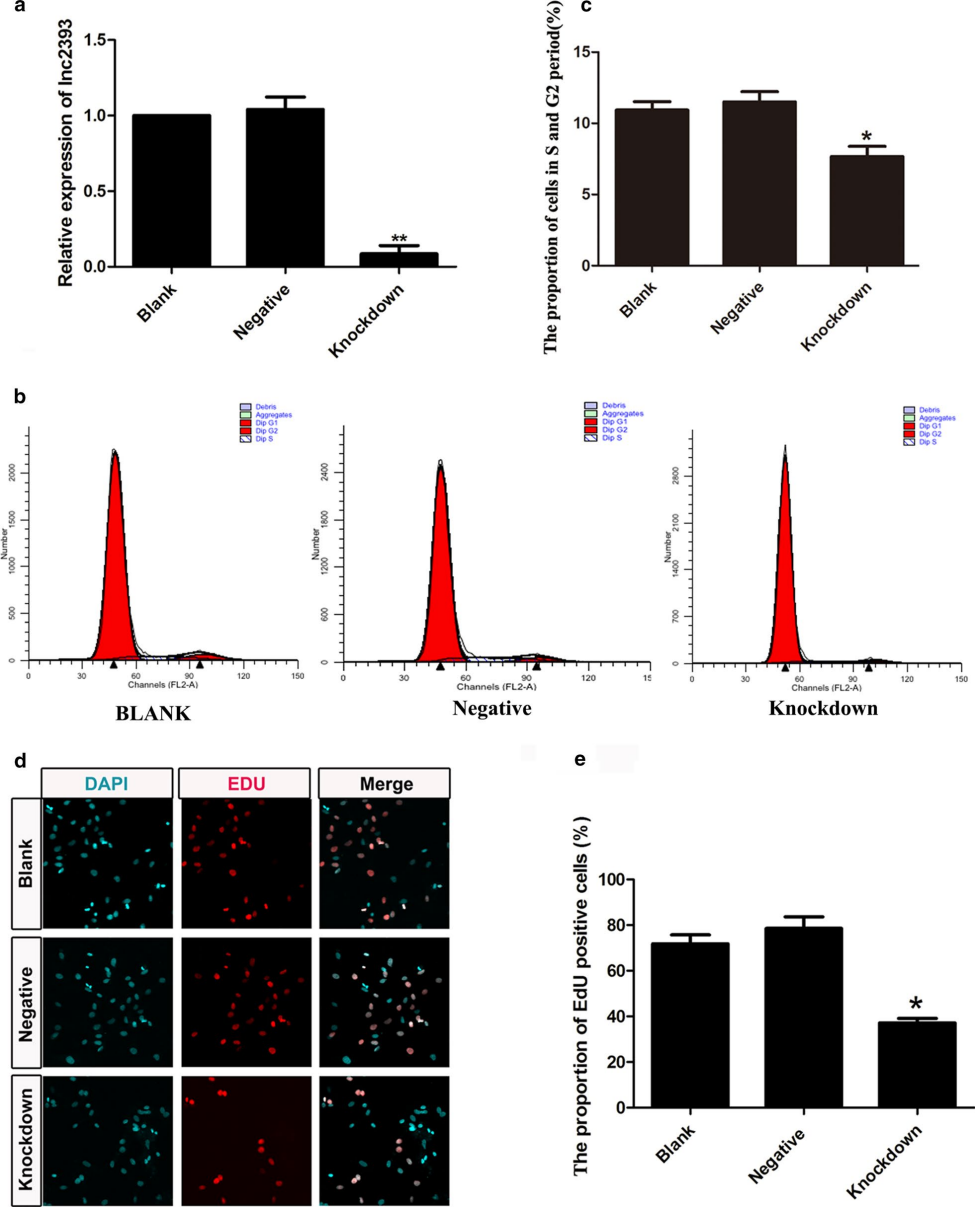
Knockdown of lncRNA2393 weakened the NSCs proliferation ability. **a** The expression of lncRNA2393 in the NSCs after knockdown compared with that in the normal NSCs (**p < 0.01). **b** Flow cytometry showed that the proliferating cells decreased. **c** Statistics analysis on the proportion of cells at G2 and S stage about flow cytometry (*p < 0.01). **d** EdU indicated that after knockdown of lncRNA2393, the EdU-positive cells (*green*) decreased. **e** Analysis of the number of positive cells in each group (*p < 0.01)

The increased expression of lncRNA2393 in the denervated hippocampus suggested that lncRNA2393 might play a vital role in neurogenesis. Consistent with this, the microarray analysis demonstrated that a number of cell-cycle genes were regulated after FF transection (Fig. 3b). In line with this hypothesis, a small interfering RNA (siRNA) was used to reduce the expression of endogenous lncRNA2393 in NSCs. The efficiency was evaluated by qPCR. RT-qPCR demonstrated that the expression of lncRNA2393 in the Knockdown group was about 72% less than that in the blank group (Fig. 7a). Using flow cytometry, the proportion of the cell population undergoing proliferation was assayed with PI staining (Fig. 7b). A significant decrease in the number of proliferating cells was observed in the lncRNA2393-depleted cells compared with the Negative group (*p* < 0.01) (Fig. 7c). siRNA-mediated knockdown of lncRNA2393 was performed under similar conditions, followed by the EdU proliferation assay to further examine the impact of lncRNA2393 on proliferation. Compared with the blank and negative control groups, the Knockdown group showed a lower percentage of cells undergoing proliferation (Fig. 7d). Analysis about the proportion of EdU positive cells showed a significance decreased in the Knockdown group. Collectively, the results indicated that lncRNA2393 reduced the proliferation of NSCs.

## Discussion

A previous study showed that the inner microenvironment changed when the FF transection was performed and the projection of basal forebrain cholinergic neurons to the DG was blocked [37, 38]. Moreover, the proliferation, differentiation, and migration of NSCs in the hippocampus are under the control of extracellular and intracellular signaling pathways. It has been observed that the changed microenvironment plays a stimulatory role in the survival and differentiation of the anterior subventricular zone (SVZa) progenitor cells after FF transection [39]; however, the underlying mechanism is not well known. Previously, researchers paid attention to the genes coding for biologically active macromolecules, including neural growth factor, brain-derived neurotrophic factor [40], and transcription factors Nng2, Mash1, Lhx8, and Brn4 [41–44].

With technological advancement and development of new-generation sequencing technology, accumulating evidence indicated that mammalian genomes encode different kinds of lncRNAs [45–47]. More and more lncRNAs were demonstrated to have biological significance in the developing nervous system through both loss- and gain-of-function experiments, either in stem cells or in vivo. Ramos and his colleagues identified and predicted over 12,000 novel lncRNAs in the subventricular zone of adult mice [48, 49]. Many lncRNA genes have now been identified, but the function and regulation of these transcripts are still unexplored.

It has been observed that the changed microenvironment plays a stimulatory role in the survival and differentiation of the SVZa progenitor cells after FF transection [39]. Hence, the question is whether this is due to the effect of lncRNAs. The relationship between the change in the expression of lncRNAs that occurs with the change in the inner microenvironment resulting from FF transection, and the neurogenesis in the hippocampus needed to be investigated further.

Therefore, microarray expression profiles were used to monitor the expression of lncRNAs after the inner microenvironment of the hippocampus changed. Using microarray and KEGG pathway analyses, 103 differentially expressed lncRNAs were identified and differentially expressed mRNAs were found to be mainly enriched in the pathways involved in infection, cell cycle, and neural development. These data indicated the possibility of the involvement of long noncoding transcripts in the gene regulation network.

Considering that the expression of lncRNAs was under the control of extracellular and intracellular signaling pathways, real-time PCR and RT-PCR were performed to testify the expression profile and tissue specificity of lncRNAs. Of these, lncRNA2393 gained the attention of researchers due to its continued increasing expression and high tissue specificity. Based on the expression profile and tissue specificity, lncRNA2393 was used for intensive studies. FISH in a frozen section of hippocampus demonstrated that lncRNA2393 existed in the infragranular layer of hippocampus and was enriched in the denervated hippocampus. Also, the relationship between lncRNA2393 and neurogenesis was investigated. The expression and location of lncRNA2393 were detected in the hippocampus-derived NSCs. Hence, lncRNA2393 existed in the NSCs and was located in the cytoplasm. Altogether, these results indicated that lncRNA2393 might be the stimulatory molecule in the hippocampal microenvironment. Loss-of-function experiments showed a significant decrease in the self-renewal of NSCs, which was consistent with the previous study results and assumption. Hence, the role of lncRNA2393 was identified as a stimulatory molecule in the hippocampal neurogenesis.

The results showed that the subcellular localization of lncRNA2393 was mainly in the cytoplasm. Previous reports indicated that the cytoplasmic lncRNAs worked mainly by competing with endogenous RNAs, for example, microRNAs, mRNAs, and pseudogenes. In particular, lncRNAs compete with these mRNAs and pseudogenes that share similar miRNA response elements to bind to the same miRNA, eventually implementing the spatial and temporal control of gene expression by preventing miRNA binding to the target genes. A study in a mouse model of Melanoma [50] demonstrated that virtually all types of RNAs could communicate with each other using MREs (MicroRNA response elements, MRFs) as the mode of communication. A study of ceRNAs in the Alzheimer’s disease showed that *BACEl*-*AS* (BACEl-antisense, BACEl-AS) could be an endogenous competitive RNA to target miRNA (mir01273, mir-1285, and mir-3064) and activate the transcription of SERFla (small EDRK-rich factor 1A, SERF1a). The upregulated expression of SERFla would further promote the aggregation of Aβ. Thus, BACEl-AS is supposed to play an important role in the pathogenesis of Alzheimer’s diseases.

Furthermore, the increased expression of lncRNA2393 was found to be triggered by the changed microenvironment. A cold environment can induce the expression of lncRNA COOLAIR, and DNA damage can induce the expression of PANDA [51]. Therefore, the question is exactly which part of the denervated hippocampus induces the expression of lncRNA2393. It needs to be testified whether some genes, such as *Sox2* and *Brn4*, are involved or other protein signals and cytokines interact with lncRNA2393. Further studies should aim to explore the molecular mechanism behind the expression of lncRNA2393 in the hippocampus, to lay the foundation for the clinical application of NSCs intreating of diseases of the central nervous system.

## Conclusion

We concluded that expression changes of lncRNAs exists in the microenvironment of denervated hippocampus, of which promotes hippocampal neurogenesis. The identified lncRNA lncRNA2393 expressed in neural stem cells, located in the subgranular zone of the dentate gyrus, which can promote NSCs proliferation in vitro. Therefore, the question is exactly which part of the denervated hippocampus induced the expression of lncRNA2393. Further studies should aim to explore the exact molecular mechanism behind the expression of lncRNA2393 in the hippocampus, to lay the foundation for the clinical application of NSCs in the treatment of diseases of the central nervous system.

## Additional file

**Additional file 1: Table S1.** Information about purity and integrity of the RNA samples. Details information about purity and integrity of the RNA samples used in the microarray, which showing all the samples have passed the quality control. **Figure S1.** Information of DNA contamination. The figure showed the result of DNA contamination check. **Figure S2.** Results of 20 candidate lncRNAs primers. Using qPCR, the proper primer which matched with the length and showed high signal value was selected from three pairs of them. **Table S2.** Summary of lncRNAs randomly selected from the microarry result. Table S2 listed the ID, change ratio and transcription ID of the differentially expressed lncRNAs. Red means the increased lncRNAs after FF transection, while the black means the decreased lncRNAs.

## Abbreviations

LncRNA: long noncoding RN
NSCs: neural stem cells
V-SVZ: ventricular–subventricular zone
DG: the dentate gyrus
miRNAs: microRNAs
FF: fimbria-fornix
RIN: RNA integrity number
cDNA: complementary DNA
PCR: polymerase chain reaction
GAPDH: glyceraldehyde-3-phosphate dehydrogenase
EGF: epidermal growth factor
bFGF: basic fibroblast growth factor
FISH: fluorescence in situ hybridization
SSC: saline sodium citrate
EdU: (5-ethynyl-2′-deoxyurdine)
PI: propidium iodide
qPCR: quantitative real-time reverse-transcription PCR
ceRNAs: competing endogenous RNAs
siRNA: small interfering RNA
SVZa: anterior subventricular zone
Ngn2: Neurogeni-2 protein
Mash1: mammalian achaete homologue-1
Lhx8: LIM homeobox 8
Brn4: brain-4
MRFs: microRNA response elements
