# Supplementary material for: Microarray expression profiling in the denervated hippocampus identifies long noncoding RNAs functionally involved in neurogenesis
Source: BMC Mol Biol. 2017 Jun 6;18:15. doi: 10.1186/s12867-017-0091-2 (PMC5461768; doi:10.1186/s12867-017-0091-2)
Supplement: Supplementary file 1 — Additional file 1: Table S1. Information about purity and integrity of the RNA samples. Details information about purity and integrity of the RNA samples used in the microarray, which showing all the samples have passed the quality control. Figure S1. Information of DNA contamination. The figure showed the result of DNA contamination check. Figure S2. Results of 20 candidate lncRNAs primers. Using qPCR, the proper primer which matched with the length and showed high signal value was selected from three pairs of them. Table S2. Summary of lncRNAs randomly selected from the microarry result. Table S2 listed the ID, change ratio and transcription ID of the differentially expressed lncRNAs. Red means the increased lncRNAs after FF transection, while the black means the decreased lncRNAs. [file 12867_2017_91_MOESM1_ESM.docx]

**Table S1.** Information about purity and integrity of the RNA samples.

| Item | Sample Name | OD260/280  ≥1.5 | OD260/230  ≥1 | Total  Amount | RIN  ≥7 | Small RNA fraction | | QC Result  ( Pass or Fail) | |
| --- | --- | --- | --- | --- | --- | --- | --- | --- | --- |
| 1 | Normal 1 | 1.88 | 2.15 | 47.78 | 7.6 | | N | Pass |  |
| 2 | Normal 2 | 1.88 | 1.98 | 32.00 | 7.6 | | N | Pass |  |
| 3 | Normal 3 | 1.88 | 1.95 | 40.61 | 7.2 | | N | Pass |  |
| 4 | Treatment 1 | 1.88 | 2.00 | 42.51 | 8.1 | | N | Pass |  |
| 5 | Treatment 2 | 1.89 | 2.08 | 38.93 | 8.1 | | N | Pass |  |
| 6 | Treatment 3 | 1.88 | 2.12 | 35.48 | 7.9 | | N | Pass |  |


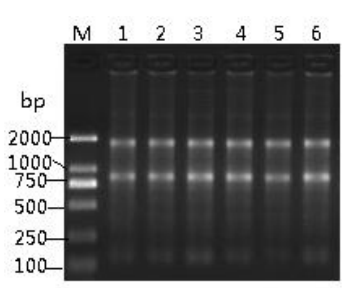


**
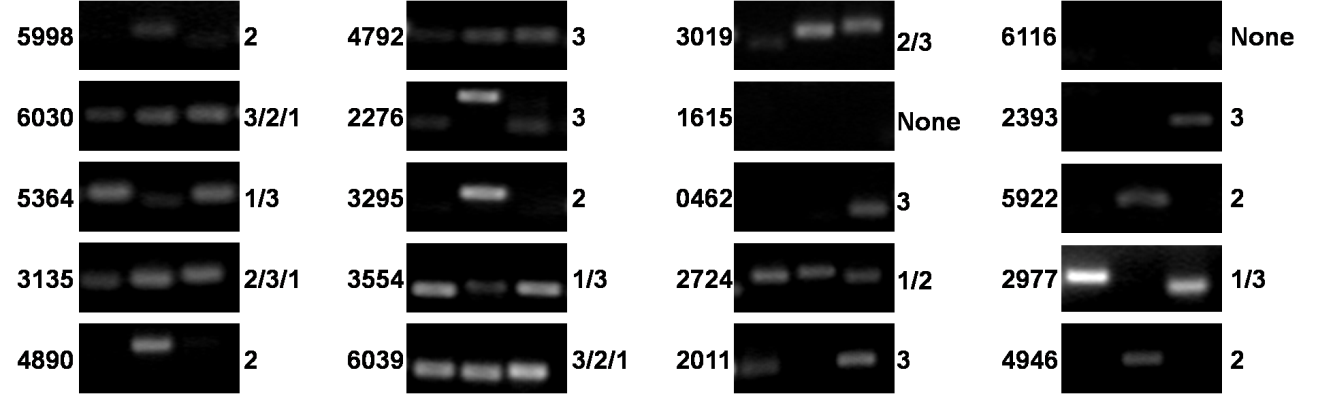
Figure S1**.Information of DNA contamination

**FigureS2** Results of 20 candidate lncRNAs primers.

Table S2 **Summary of lncRNAs randomly selected from the microarry result.** Red means the increased lncRNAs after FF transection, while the black means the decreased lncRNAs.

| ID | Ratio | transcription_ID |
| --- | --- | --- |
| RB_p_rnol003019 | 7.022697359 | ENSRNOT00000037631 |
| RB_p_rnol002977 | 2.765922921 | ENSRNOT00000035283 |
| RB_p_rnol002393 | 3.167740533 | ENSRNOT00000036611 |
| RB_p_rnol002724 | 2.071017274 | ENSRNOT00000038344 |
| RB_p_rnol003135 | 2.117569787 | ENSRNOT00000073813 |
| RB_p_rnol005364 | 2.553447248 | ENSRNOT00000052065 |
| RB_p_rnol006030 | 2.155873896 | ENSRNOT00000071854 |
| RB_p_rnol005998 | 3.268128917 | ENSRNOT00000075272 |
| RB_p_rnol005922 | 2.833333333 | Mouse_homology_transcript(2929):NR_033620 |
| RB_p_rnol004890 | 2.091362811 | ENSRNOT00000009721 |
| RB_p_rnol003295 | 3.556863803 | Mouse_homology_transcript(2772):NR_030673 |
| RB_p_rnol003554 | 2.211021154 | ENSRNOT00000027501 |
| RB_p_rnol004792 | 2.036483021 | ENSRNOT00000047322 |
| RB_p_rnol006039 | 2.321195652 | XR_147166.1 |
| RB_p_rnol000462 | 2.317794486 | Mouse_homology_transcript(1316):NR_033745 |
| RB_p_rnol006116 | 2.126764706 | Mouse_homology_transcript(274):NR_045744 |
| RB_p_rnol001615 | 2.087113518 | Mouse_homology_transcript(1584):NR_102387 |
| RB_p_rnol002011 | 2.227689741 | Mouse_homology_transcript(3150):NR_045507 |
| RB_p_rnol002276 | 2.174579985 | Mouse_homology_transcript(1441):NR_046002 |
| RB_p_rnol004946 | 2.007826768 | Mouse_homology_transcript(1191):NR_028126 |
